# An Initial Analysis of the Effects of a Long-Term Care Insurance on Equity and Efficiency: A Case Study of Qingdao City in China

**DOI:** 10.1177/0164027520907346

**Published:** 2020-02-25

**Authors:** Wei Yang, Shuang Chang, Wenbo Zhang, Ruobing Wang, Elias Mossialos, Xun Wu, Dan Cui, Hao Li, Hong Mi

**Affiliations:** 1 Department of Global Health & Social Medicine, 4616 King’s College London, United Kingdom; 2 Department of Computer Science, 13290 Tokyo Institute of Technology, Japan; 3 Department of Social Policy, Institute of Sociology, 12470 Chinese Academy of Social Sciences, Beijing, China; 4 London School of Economics and Political Science, United Kingdom; 5 The Hong Kong University of Science and Technology, Kowloon, Hong Kong; 6 Global Health Institute, 12390 Wuhan University, China; 7 12377 Zhejiang University, Hangzhou, China

**Keywords:** long-term care insurance, China, equity, efficiency

## Abstract

Finding a suitable mechanism to finance long-term care (LTC) is a pressing policy concern for many countries. Using Qingdao city in China as a case study, this article presents an initial assessment of a newly piloted LTC insurance by evaluating its effects on equity and efficiency in financing. Drawing data from 47 in-depth interviews conducted in 2016, this study found that there remain sizable disparities in financial burden among insurance participants, despite an emphasis on ensuring equitable access to care. Although the insurance brought cost savings to the health care sector, the LTC providers are incentivized to provide care at the least cost, even when such care is deemed inadequate due to the fixed payment for their services. This article offers critical insights into the potentials and challenges of applying the LTC insurance model in a developing country, where critical lessons can be drawn for public LTC insurance in other countries.

Population aging has created an unprecedented global challenge: The number of older adults is expected to grow from an estimated 524 million in 2010 to nearly 1.5 billion by 2050, with most of this increase taking place in low- and middle-income countries (LMICs; World Health Organization, 2015). As a result, the demand for responsive and quality long-term care (LTC) has risen rapidly in both developed and developing countries (Costa-Font & Courbage, 2012; X. Zhang et al., 2018), and the financing of such care has become an increasingly important policy issue for many countries’ governments. Several countries have chosen to use a social LTC insurance model to fund their LTC systems. Researchers argue that this model offers many distinct advantages over the two major alternatives, namely the tax-based model and the means-tested model (Launois, 1996). Although the social LTC insurance model is gaining popularity (Chon, 2013; Costa-i-Font, 2011; Nadash & Shih, 2013; Rhee et al., 2015; Zuchandke et al., 2012), to date, only five high-income countries (i.e., Germany, the Netherlands, Luxemburg, Japan, and South Korea) have adopted it. Evidence on whether this model can achieve similar goals in LMICs, where insurance benefit packages can be limited due to a shortage of revenue from public funds, is scant.

China, with the total number of older adults (aged 65 and above) reaching 110 million or 8% of the population, is among the first LMICs to pilot a social LTC Insurance program (National Bureau of Statistics of P.R.China, 2011). Officially piloted in 2012, Long-Term Care Nursing Insurance (LTCNI) is a dedicated insurance program that covers professional LTC and geriatric services for those with substantial or critical LTC needs. Early assessments of the pilot scheme have yielded mixed results (Qin et al., 2014; Yuan, 2013; Zhao, 2015). Some studies found that the scheme has made services more affordable for participants (Deng & Guo, 2015) and has generated substantial savings for social health insurance (SHI) funds as the fixed per diem rate for LTC accounted for a smaller fraction of the average costs of inpatient care in hospitals (Lv & Wu, 2016). Other studies reported discrepancies in the reimbursement rates for services for participants with different SHI status (An et al., 2017) and poor coverage for those with no formal employment or living in rural areas (Yang et al., 2017; W. Zhang, 2017).

Previous studies have contributed to a preliminary understanding of the impacts of LTCNI on equity and efficiency, but the limited available evidence remains descriptive in nature. This article seeks to bridge the gap by providing an initial empirical assessment of the performance of LTCNI by evaluating its impact on equity and efficiency in financing. We use Qingdao—the first city that piloted LTCNI in 2016—as a case study. The study draws its data from semistructured in-depth interviews.

## Public LTC Insurance Model in China and Other Countries

Several high-income countries, including Germany, the Netherlands, Luxemburg, Japan, and South Korea, use a stand-alone, dedicated insurance program for LTC services. In the above countries, LTC insurance is predominantly financed through an employment-based, payroll contribution. Participation in the LTC insurance is mandatory for either the whole population or just a segment of it. Apart from Japan, where only those aged 40 and above are expected to pay the premium, the countries’ adults of working age (or retired population in some countries) need to pay a mandatory premium for their LTC insurance. As a result, the coverage usually reaches the entire population who need care. The scope of covered services varies in different countries, but all include dementia care. In some countries, lodging costs in nursing homes can also be covered, subject to individual co-payments. Except for South Korea, countries separate their LTC insurance programs from their SHI programs in terms of risk pooling, revenue collection, and administration. Table 1 summarizes the key features of the LTC insurance programs in Germany, the Netherlands, Luxemburg, Japan, and South Korea.

**Table 1.** Key Features of the LTC Insurance Programs in Germany, Luxembourg, the Netherlands, Japan, and South Korea.

Country	Year of Inception	Eligibility	Premium	Benefits	Cognitive Impairment
Germany	1995	Disabled older people assessed as needing LTC, no age restriction	Payroll contributions by all working-age and retired population, employers also need to contribute proportionally	Fixed in-kind or cash payments depending on the individual’s preference and need for home-based care, fixed monthly payments depending on the need for nursing home care. Approximately 80% of the care costs can be covered	Covered
Luxembourg	1999	Disabled older people assessed as needing LTC, no age restriction	Payroll contributions by all working-age and retired population	In-kind and/or cash, depending on the individual’s preference	Covered
The Netherlands	1968	Disabled older people assessed as needing LTC, no age restriction	Payroll contributions by all working-age and retired population	In-kind for institutional care and cash for home care.	Covered
Japan	2000	Disabled older people, aged 65 and above needing LTC, or disabled people, aged 40 and above, with age-related disabilities	Payroll contributions for individuals aged 40 and above	In-kind, about 90% of the care costs can be reimbursed	Covered
South Korea	2008	Disabled older people, aged 65 and above needing LTC, or disabled people with geriatric diseases	Payroll contributions by all of working age to the health insurance scheme	In-kind or cash depending on the individual’s needs	Covered since 2016

*Note.* LTC = Long-term care; LTNCI = Long-term Care Nursing Insurance. *Source.* Campbell et al. (2010), Costa-i-Font (2011), Kobayashi and Reich (1993), Matsuda and Yamamoto (2001), Rhee et al. (2015), Schut and van den Berg (2012), and Zuchandke et al. (2012).

In comparison with the insurances in the countries mentioned above, LTCNI in Qingdao is much more limited in terms of its level of covered population and benefits. China piloted its first LTC insurance program in Qingdao in 2012. Since its inception, LTCNI in Qingdao has expanded its coverage, and the total number of beneficiaries reached 32,056 by the end of 2015, of which around 55% were female. The total number of beneficiaries accounts for approximately 2.6% of the senior population. The average age for LTCNI participants was 77.8 years old in 2015. Table 2 shows the age distribution of LTC insurance beneficiaries. The majority of the beneficiaries were 80 years old and above (Zhejiang University, Qingdao Human Resource and Social Security Bureau, & Qingdao Finance Bureau, 2018).

**Table 2.** Age Distribution of LTCNI Beneficiaries.

Age groups	2012 (%)	2013 (%)	2014 (%)	2015 (%)
<60	5.5	6.0	5.9	8.1
60–70	9.1	9.2	9.2	12.6
70–80	29.0	26.8	24.6	28.4
80 and above	56.4	58.0	60.3	50.9

*Note.* LTNCI = Long-term Care Nursing Insurance. *Source.* Zhejiang University et al. (2018).

It is important to have an overview of the design features of LTCNI, which sets the scene for the analysis. The information is largely garnered from preliminary fieldwork conducted in Qingdao over the summer of 2016 and from various government policy documents (Qingdao Human Resource and Social Security Bureau et al., 2012; Qingdao Human Resource and Social Security Bureau et al., 2014). Table 3 summarizes the key features of LTCNI. It is important to note that LTCNI is consistently evolving, and this article is based on the data collected in 2016.

**Table 3.** The Main Features of LTCNI in 2016.

Revenue collection	0.5% of UEI funds and 10% of URI funds are accrued to LTCNI annually.	
Purchasing	Secondary and tertiary hospitals	RMB170 (US$24.5) per day for care provided at secondary and tertiary hospitals.
	Nursing home	RMB65 (US$9.36) per day for nursing homes.
	Home-based care	RMB50 (US$7.2) per day for home-based care.
Fund pooling	Qingdao city level.	
Services covered	Secondary and tertiary hospitals	For institutional care provided at hospitals and nursing homes, services covered include nursing care provided by designated care providers. Meals, bed fees, and carer costs are payable by the patients.
	Nursing home	
	Home-based care	Home-based care includes at least 2 weekly visits from doctors and nurses. Basic medical products, such as drugs or adult diapers, are covered by the insurance. However, LTCNI does not cover day-to-day assistance.
Eligibility	Eligible participants need to satisfy the following criteria. They must be Qingdao residents who are enrolled in the UEI or the URI, have completed a 10-item needs assessment and have been identified as having substantial/critical needs, have received a diagnosis from hospital staff or care providers confirming that they suffer from at least one of the listed conditions covered by LTCNI, and have incurred annual health-care expenses of RMB5,000 (US$720.2) or more for the 2 previous years.	
Premiums and co-payments	UEI	The reimbursement rate is set at 90% for older people who are enrolled in the UEI; no premiums or surcharges are required.
	URI	80% for URI participants with an annual contribution of RMB350 (US$50.4) and 40% for URI participants with an annual contribution of RMB110 (US$15.8).

*Note.* UEI = Urban Employee Insurance; URI = Urban Resident Insurance; LTCNI = Long-term Care Nursing Insurance.

### Eligibility Rules

Eligibility rules are important components of any health/LTC financing policy, as they determine the target beneficiaries. Unlike other countries, enrollment in LTCNI is tightly linked to the status of individuals’ SHI, which is made up of three SHI schemes—the Urban Employee Insurance (UEI), covering urban residents with formal employment; the Urban Resident Insurance (URI), covering urban residents without formal employment; and the New Rural Cooperative Scheme, covering rural residents. Eligible participants in LTCNI must be enrolled in either the UEI or the URI. Also, they must have critical LTC needs (often being bedridden) as determined by a 10-item Activity Daily Living (ADL) Questionnaire. A diagnosis from hospitals or care providers confirming an applicant’s condition is also required for program enrollment. Young people can be eligible if they meet the eligibility criteria; however, unlike other countries’ programs, as of 2015, older people suffering from dementia/severe cognitive impairments were not covered in the insurance.

### Services Covered

Four types of services can be provided depending on the patients’ needs and conditions: (1) hospital care at a designated secondary or tertiary hospital providing 24-hr intensive care for older adults with critical LTC needs, (2) nursing home care intended for older adults who require continual care and have substantial difficulties coping with normal ADL, (3) home-based nursing care including weekly care visits by doctors or nurses, and (4) basic home-based care provided by health visitors in rural areas, available since 2015 (Qingdao Human Resource and Social Security Bureau, 2014). As the number of eligible participants continues to grow, institutional LTC care is only available for the UEI and some URI participants (Qingdao Human Resource and Social Security Bureau et al., 2014). Approximately 87.2% of LTCNI beneficiaries used home-based services, whereas the percentage of beneficiaries that used institutional care was only 12.8% in 2015.

### Main Sources of Revenue and Service Costs

**Figure 1.**
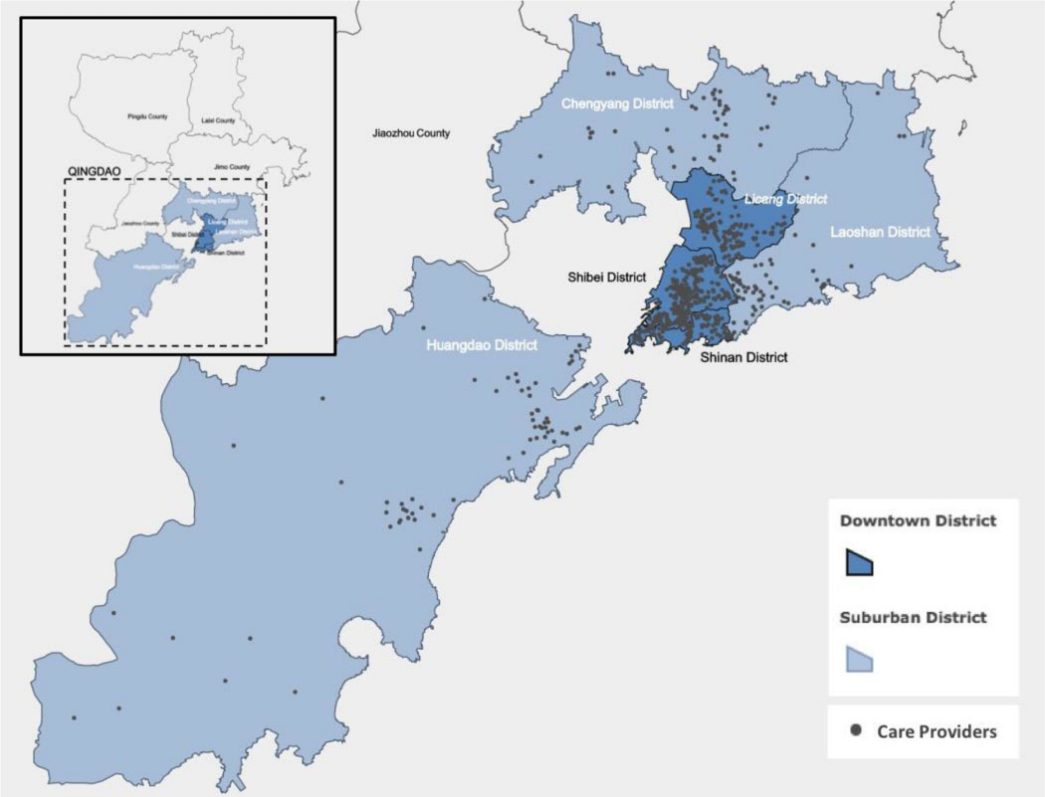
Map of the designated care providers covered by the Long-term Care Nursing Insurance in Qingdao.

In terms of financing, revenue for LTCNI has come from the UEI and the URI since 2015 (Qingdao Human Resource and Social Security Bureau et al., 2014). Purchasing prices are fixed at RMB170 (US$24.5) per day for care provided at secondary and tertiary hospitals, RMB65 (US$9.4) per day for nursing homes, and RMB50 (US$7.2) per day for home-based care. Reimbursement rates are also different for participants in different SHI schemes, ranging from 90% for UEI participants to 40% for URI participants with an annual contribution of RMB110 (US$15.8; Qingdao Human Resource and Social Security Bureau et al., 2014). Services and drugs prescribed within the LTC facilities are reimbursed under LTCNI. If these services and drugs are prescribed by doctors in health facilities, then these will be reimbursed by the senior’s SHI. Board and lodging costs have to be paid out of pocket. In 2016, Qingdao had more than 500 designated LTC providers. Figure 1 shows the locations of these providers.

## Equity in Access to Care and Efficiency in Financing

This study aims to provide an initial evaluation of LTCNI against two main policy objectives—equity and efficiency, which are two highly contentious concepts. Based on preliminary fieldwork and published peer-reviewed manuscripts on a similar topic (Yang et al., 2016), this study narrows down the assessment by looking at horizontal equity, allocative efficiency, and technical efficiency. For horizontal equity, the study seeks to know whether LTCNI has a redistribution effect of allowing resources to be distributed according to need. Although LTCNI requires a needs assessment to determine the eligibility of participants, the reimbursement rate is still based on an individual’s SHI status; hence, it is not clear whether LTCNI can alleviate the burden of those who are at risk of financial catastrophe from care costs. As the health and LTC sectors are administratively separated, it is important to assess the allocative efficiency of resources in these two sectors—revealing whether investment in the LTC sector leads to cost savings in the health sector. The “substitution effects” of LTC insurance, as evident from the literature (Forder, 2009), may lead to a reduction in hospital admissions and associated costs—a key policy goal of LTCNI. Technical efficiency is particularly relevant for the purpose of this study. Scholars have discussed the relationship between technical efficiency and prospective payment mechanisms (Barros, 2003)—a provider payment mechanism that is adopted by most social LTC insurances, including LTCNI in China. As documented in the literature, care providers tend to “skim” funds from less severe treatments and more lucrative patients if they are paid with a predetermined, fixed amount. Drawing from the discussion above, this study seeks to examine the following research questions regarding equity and efficiency:

1. What is the impact of the LTCNI on inequities in access to the LTC? (horizontal equity)
2. Does the LTCNI affect bring cost saving to health care sector by offering a per diem rate for care? (allocative efficiency)
3. Does the funds of the LTCNI been efficiently used by the care providers? (technical efficiency)

## Method

This study chose Qingdao as the case study site, as Qingdao’s experience is a critical case for the purpose of this study (Flyvbjerg, 2006). Identifying critical cases is crucial for the analysis because findings from such cases are likely to be generalizable. LTCNI in Qingdao has the most comprehensive coverage and benefits packages (e.g., target population, level of reimbursement, and covered services) among the pilot cities at the time of the study. It also introduced a needs assessment to direct resources to the neediest population and a fixed daily rate for the provider to ensure that services are delivered in the most cost-effective way. The problems revealed in this case study on Qingdao are also relevant to other pilot cities.

The fieldwork for this study took place in 2016, deploying semistructured, in-depth individual or group interviews. The study adopted a purposive sampling strategy to identify and select groups of individuals that are especially knowledgeable about LTCNI and the LTC systems in Qingdao (Marshall, 1996; Robinson, 2014). The study focused on interviews with three types of respondents: government officials at the Human Resources and Social Security Bureau (HRSSB) and the Civil Affairs Bureau (CAB) in Qingdao (*N* = 4), relevant personnel at the care provider level (i.e., executive staff at the local care facilities, frontline doctors, nurses, and care workers; *N* = 29), and the family members of service users (*N* = 14). Fourteen care providers were purposively selected to capture different types of care provision: secondary or tertiary care provider, nursing care provider, and home-based care. These care providers were located in districts with different economic developments. The family members of the service users were selected at the selected providers. Table 4 shows the general attributes of the respondents and the number of interviews conducted.

**Table 4.** Attributes and Profiles of the Respondents.

Respondents	Categories		Number of Respondents
Government officials	The HRSSB		1
	The CAB		3
Care providers	Secondary and tertiary hospitals	Executive staff	4
		Frontline staff (i.e., doctors, nurses, and care workers)	4
	Nursing homes	Executive staff	6
		Frontline staff (i.e., doctors, nurses, and care workers)	3
	Home-based care facilities	Executive staff	3
		Frontline staff (i.e., doctors, nurses, and care workers)	9
Family members of LTCNI participants	Secondary and tertiary hospitals		1
	Nursing homes		3
	Home-based care facilities		10

*Note.* HRSSB = Human Resources and Social Security Bureau; CAB = Civil Affairs Bureau; LTCNI = Long-term Care Nursing Insurance.

Thirty-one semistructured individual interviews, and two group interviews with government officials from HRSSB and CAB, were conducted to reach data saturation (Guest et al., 2006). During the interviews, the researchers used interview guides and probed for the details of information revealed by the respondents by relating to the conceptual framework. The interviews were recorded and transcribed. All interviews were conducted in a face-to-face manner and in the Chinese language. The study included coding and thematic analysis to analyze the data. All data were manually double coded. They were coded by the first author and then coded again by the second author. The researchers read through the interview data record and then identified the codes from the data. This was followed by extracting the prominent themes that emerged from the codes. These themes were discussed and agreed upon by the authors. To ensure the data are not biased, they were checked and matched with a review of key government policy documents and reports. The Results section only presents the most critical responses from the interviews, and other data verification is not presented due to the length constraints of this article.

## Results

This section discusses the three research questions relating to equity and efficiency. It will present relevant themes/results under each research question.

### Research Question 1: What Is the Impact of LTCNI on Inequities in Access to LTC?

#### Improved access to LTC among those with substantial and critical needs

One of the main positive changes brought by LTCNI is that the receipt of services is closely linked to individual needs. The eligibility of the scheme is determined by a needs assessment. As explained by a government official:

a standardized ADL questionnaire is used to assess eligibility…the patient is assessed by a doctor or nurse at a designated care provider…only those who are under 60 points, usually bedridden, can participate in the scheme.

In addition to this preliminary assessment, “a secondary assessment that involves video recording the applicant is required to show the physical functionality of the patient.” A government official from HRSBB also stated that “when we cannot make a decision by watching the video of the second assessment, our medical team will visit the applicant in person to assess their needs for nursing care and other related treatments.” As noted by one government official from the CAB,

We have more than 120, 000 disabled people in Qingdao, but only half of the people have received some LTC…It is important to identify the neediest population…By introducing the needs assessment score system, we can target our resources to them. We started by piloting a very simple needs assessment in a few districts. Now we are using a uniform needs assessment form across the city.

A care home staff member also confirmed the needs assessment process:

(These patients) are disabled patients, such as stroke patients…They have to go through a needs assessment. Those who score under 60 are eligible to apply (for LTCNI-funded services)…The CAB will also visit patients who sign up for home-based care services to re-check the level of their needs. They usually visit the patients without informing us (to make sure they patients are not exaggerating or faking).

#### Disparities in benefit packages among LTCNI participants with different SHI status

Although the fact that the individual’s LTC needs have been taken into account when accessing publicly funded services, our data showed that, as LTCNI primarily draws its funds from different SHI schemes, the membership and benefits packages can significantly differ, even among participants that have the same level of LTC needs. As shown in Table 1, the reimbursement rate is fixed at 90% for the UEI participants. In contrast, the rates are much lower for the URI participants—80% and 40% for those enrolled in the URI with an annual contribution of RMB350 (US$50.4; URI Category A) and RMB110 (US$15.8; URI Category B), respectively. It is also notable that those enrolled in the URI Category B are not eligible for any institutional care (care provided at secondary and tertiary hospitals and nursing homes); only basic home-based care provided by health-care visitors is available to those participants.

As a result of the differences in the benefit packages, a large proportion of service users at secondary and tertiary hospitals and nursing homes are the UEI participants—those with formal employment. Noted by an executive staff member at a nursing home: “We only have 2 URI participants, the rest (29 patients) are all UEI participants.” Similar accounts were provided by the staff at care homes: “We have a very small number of URI participants…almost all patients are enrolled in UEI…Out-of-pocket payments are still considered high for URI participants.”

Some family members of URI participants also expressed discontent toward the high co-payments:

…My mother has to pay more than 40% (of the total care costs) out of pocket…This is a serious issue because many older people were once housewives or have never had a formal job…they do not have pensions…the government needs to implement some policies for these people. (Family member of the older person)

We pay around RMB2,400–2,500 (US$345.7–360.1) out of pocket per month…In winter, there are extra costs for heating…My family can still afford these costs, but it would be very difficult for some people, for example, those former factory workers who were laid off because the factories they worked in were closed. Their pension is just around RMB1,000 (US$144.04) per month…There are a lot of them in Qingdao…Overall, I think the costs of care are still high. (Family member of the older person)

Narratives from the respondents showed that a significant degree of inequity exists in the distribution of the cost burden among different SHI participants. In particular, the URI participants, who usually have had no formal employment, are more likely to incur high care costs than the UEI participants.

#### Poor access to care for those with cognitive impairments

Another problem noted in the interviews is that LTCNI does not cover older people with cognitive impairments unless they meet the requirements of the needs assessment (a needs assessment score under 60 or bedridden). According to a government official at HRSSB, the main reason for not covering these older people is the high care costs associated with managing people with cognitive impairments:

They (older adults with moderate to severe cognitive impairments) often require around-the-clock care and supervision. We will have to have someone look after them all the time. If they get lost or hide somewhere, and we cannot find them, the costs can be tremendous…

Interview data suggested that “around 15% of older people aged 60 and above may suffer from cognitive impairments” (Government official). However, those who do not pass the needs assessment, which only assesses physical functionality, are excluded from the scheme (Government officials).

### Research Question 2: Does LTCNI Facilitate Cost Savings in the Health Care Sector by Offering a Per Diem Rate for Care?

The LTC system usually works in close step with the health care system. Without an established LTC system, hospital care often becomes the first point of contact for older people needing LTC. Older adults not only undergo unnecessary treatments during hospitalization but also worry that hospital care for a long duration can result in high medical bills. One of the key aims of LTCNI is to reduce hospital admissions of older people and their length of stay in hospitals when their acute care phase is over. According to government officials from the HRSSB:

From 2014 to 2015, LTCNI has spent just under RMB91 million (US$13.11 million) (for the care). This money covered more than 40,000 older people. If these patients were to use hospital services, each visit could cost the SHI a few thousands or even tens of thousands. LTCNI is a very efficient method to reduce care costs.

As a per diem rate for care is agreed upon by LTCNI and the designated care providers, these rates are much lower compared to the cost of equivalent care provided at hospitals. LTCNI participants only pay a fixed cost for their care, and this also means a significant reduction in their care costs.

(In the nursing home,) we only pay for the drugs controlling his seizures, plus the costs for staying here…The costs were much higher when he was hospitalized…He stayed (in the hospital) for two or three nights, and that cost us more than RMB20,000 (US$2880.77). (Family member of a senior)

The per diem cost at the intensive care unit can be as high as RMB5000 (US$720.2). If the condition of the older person is stable, they can be transferred to a designated secondary or tertiary care provider. The provider can offer very similar care (as what the patient receives in the hospital) but at a much lower cost—around RMB170 (US$24.5) per day…We think that LTCNI helps a lot to reduce the financial burden on patients. (Staff from a nursing care provider)

In addition to bringing cost savings to the health care sector, LTCNI has also reduced the demand for care homes by encouraging the use of home-based LTC. For instance, in 2013, LTCNI started to include more home-based services such as on-call services and longer hours of home visits (Qingdao Human Resource and Social Security Bureau et al., 2014). This change not only addresses the preference of the Chinese people to receive care at home but also limits the cost of institutional care, which causes a high financial burden on families. As noted by a respondent:

He cannot move, and it is very difficult to take him to the hospital, even for regular check-ups. With weekly doctor visits, things are much more convenient. The doctors and nurses are on call all the time. We do not need to go to the hospital all the time anymore. This saves us money and energy. (Family member of a senior)

A home-based care provider described the process of how they teach the family members to manage some care conditions at home:

Previously many disabled older people frequently went to hospitals (when they had problems). Sometimes, what these people need is not hospital care but LTC…Now we have LTCNI, which means that older people can receive these services at home. We also provide some basic training to family members, so they can help the patient manage many of their medical conditions at home. (Staff from a home-based care provider)

The narratives from the respondents indicated that LTCNI plays an active role in cost containment and the improved allocative efficiency of care delivery.

### Research Question 3: Have the Funds of LTCNI Been Efficiently Used by Care Providers?

#### Skimming behavior among care providers

Although LTCNI has improved allocative efficiency, some providers are incentivized to scrutinize potential service users to assess whether or not their expected care costs would exceed the fixed cost and to reject applications where this is the case. As stated by a government official: “Some providers may find excuses to deter users who are likely to incur high costs, or they may deliver less than needed of care to patients with severe LTC needs” (Government official). As noted by an informant from a care provider:

We are paid at a fixed cost. This means that we have to have some control over which patients we admit. We will definitely lose money if we keep admitting the older adults with severe disabilities…For example, when an older person constantly gets infections and needs antibiotics to control them, the cost can easily go up to RMB300–500 (US$43.21–72.09) per day…We cannot accept these patients, and we suggest that they be transferred to hospitals. (Staff from a secondary and tertiary care provider)

As the providers are paid with flat rates, providers may end up running a deficit if they admit a larger group of severely disabled users. Further, service users may not always receive appropriate treatment. For example, interviews revealed that some providers tend to be “very careful with the medicines they prescribe and the services they provide because of the associated costs” (Staff from a secondary and tertiary care provider). Other respondents mentioned that they tended to prescribe less expensive medicines to contain costs: “We have to have strong measures to control the costs. If we can use generic medicines, we will not use patented ones, because the cost differences are usually huge” (Executive staff from a home-based care provider). As stated by an informant at a nursing home:

Some providers are inclined to prescribe relatively low-cost medicines. For example, some cheap anticoagulant medicines only cost RMB0.3 (US$0.043) per unit, whereas the expensive ones can be 10 times as expensive. Certain providers will only use the cheap ones without considering the clinical efficacy of the medicines. (Staff from a nursing home)

Similar accounts were provided by a home-based care provider:

Many patients need incontinence pads, but we have to control the use. If they use a lot, the costs will increase and will be too expensive for us. I have to say, even though older adults are covered by LTCNI, we cannot give whatever they need. We have to control what we prescribe and what we offer, in order to control the costs. (Staff from a home-based care provider)

The narratives show that, as payments do not vary according to the user’s dependency level, providers are likely to skim from treating severely disabled users and the underuse of services. Technical efficiency can be undermined because resources are directed to the less severe patients because of cream skimming.

#### Lack of information on entitlement to services and eligibility rules for potential users

One key issue to avoid skimming is to ensure that adequate information on entitlement and eligibility is available to the public. However, the interviews showed that patients are not always informed of their eligibility for enrollment in LTCNI or their entitlements to funded services.

We do not know the criteria (for receiving the services). We just applied, and they decided whether we can receive the services or not. It seems that you have to be paralyzed (in order to be eligible). But we are not sure…(We) do not know whom we can ask for this information. (Family member of an older person)

Other participants also confirmed that most of them only found out about LTCNI through *friends or relatives*, but most of them were *not clear about the eligibility* of receiving funded services. Some eligible patients were even rejected by care providers, even though they are entitled to the services. A care home manager mentioned a case of an old patient:

She had been to many (providers). She satisfied all the criteria for receiving funded services…But nobody wanted to take her…She was very sick and full of piles…The competition in the care home marketing is intense, and the cost (of taking care of severely disabled patients) is very expensive. We admitted her to our nursing home, but honestly, we do not make any money off of her.

The potential service users are often unclear about what services they are entitled to, which can lead to unequal or unfair treatment. The case of the older patient mentioned above illustrates how an eligible patient can be rejected by providers. This could potentially be avoided if the patient were given adequate information on their entitlements.

## Discussion and Conclusion

This article provides an initial assessment of LTCNI—China’s first LTC insurance scheme—focusing on equity and efficiency. It is among the first studies to evaluate social LTC insurance in an LMIC, and China’s experience of LTC financing will offer useful lessons for other LMICs with similar demographic structures. The study highlights several specific issues with the scheme and proposes three policy recommendations to address them.

### Improving the Benefits Package of LTNCI for Those Who Are From Lower Socioeconomic Groups

A key aspect of LTC financing based on an equity criterion is that a care-dependent person should not be denied access to care when they are unable to meet the costs. LTCNI, however, is regressive among the participants: The URI participants who are comparatively poorer are more likely to incur higher co-payments or encounter access barriers because of their low reimbursement rate compared to the UEI participants. While a universal or more comprehensive financing system may provide a potential solution to the problem, in the long run, funding can also be targeted to satisfy the needs of the lower socioeconomic groups who are at risk of incurring higher than expected costs.

### Implementing a Risk-Adjusted Provider Payment Mechanism Based on Patient LTC

While LTCNI might be able to contain costs by negotiating a fixed price with care providers, our results also revealed that such payment arrangements allow providers to practice risk selection, leaving the most vulnerable people uncovered. This phenomenon has been documented in other publicly funded health or LTC systems (Feng et al., 2011; Fernandez & Forder, 2012; Hsiao, 2007). To address this issue, a risk-adjusted payment mechanism, whereby providers receive higher payments for patients with higher needs, can be potentially helpful in diminishing the incentives for risk selection. This should also be accompanied by a formalized system to facilitate quality monitoring and regulatory oversight (Colombo et al., 2011).

The analysis of the performance of LTCNI in Qingdao points to several inherent trade-offs among key objectives in financing LTC through an insurance-based model. For instance, the government has the fiduciary responsibility to ensure that the money spent on LTC is cost-effective, a principle concern for China and many other countries, but care providers may be incentivized to provide care at the least cost, even when this care is deemed insufficient or inadequate, which could then lead to significant consequences for patients’ well-being.

### Introducing Hypothecated Premiums and Pooling Funds Specific to LTCNI

Self-evidently, an LTC system needs to be financially solvent and affordable for the public purse. The rollout of a national LTC insurance program in China means that the number of potential beneficiaries may grow at a more rapid rate than the underlying aging population growth—a phenomenon that has occurred in many other countries following the launch of a universal LTC program (Fernandez & Forder, 2012). Using the SHI fund to finance LTCNI may not be a viable long-term option, and there is a need to institute a financing mechanism to supplement SHI funding with hypothecated premiums and pooling funds specific to LTC services. In Germany, Japan, and South Korea, the compulsory public LTC insurances require mandatory premium contributions from employed people of working age (Chon, 2014; Tamiya et al., 2011; Zuchandke et al., 2012). Allowing a degree of defined contributions is important to guarantee the financial suitability of an LTC insurance system.

This study has a number of limitations. First, due to the scope and time frame of the study, it is not feasible to collect quantitative data. Although the interview data have provided useful insights on issues relating to the equity and efficiency of LTCNI, this study could have been strengthened by incorporating more quantitative data, which were not publicly available at the time of the analysis. Second, the analysis is based on interviews from 47 key informants in Qingdao city. Qingdao’s experience may be representative of insurance programs in other cities with a similar design, but the generalization of the study results to the whole of China should be taken with caution as Chinese regions vary significantly in terms of economic development and demographic structures. Third, the above section presents an initial policy evaluation of LTCNI in China. However, it is important to understand that some contextual factors, such as cultural issues or the fiscal capacity of the local government, are not empirically assessed in the analysis.
